# Frontal Sinus Barotrauma

**DOI:** 10.5334/jbr-btr.908

**Published:** 2016-04-27

**Authors:** Ruben Vandenbulcke, Bartel van Holsbeeck, Ilse Crevits, Jesse Marrannes

**Affiliations:** 1 AZ DELTA, Roeselare, BE

**Keywords:** Barotrauma, frontal sinus, submucosal hematoma

A 58-year-old woman visited our hospital’s emergency department because of a severe and increasing pain over the right occipital region, irradiating over the right frontal area. The pain started during the descent of a jet airplane on a holiday trip. There were no visual or hearing deficits, head trauma, or epistaxis, and she experienced no difficulty clearing her ears during the flight. Only a minor respiratory tract infection was noted two weeks before this episode, and she had no history of sinus problems. The pain was nothing like previous migraine headaches she had experienced.

A noncontrast CT scan was performed which showed no apparent abnormal findings, except for a soft tissue density in the right frontal sinus, initially interpreted as an inflammatory mucosal swelling. Because of the atypical and persistent headache, MRI imaging was performed to exclude underlying pathology. No intracranial mass, hemosiderin deposits, or arteriovenous malformation were observed. A polypoid mass was noted in the right frontal sinus, hyperintense on T1- and T2-weighted images, without enhancement after contrast administration (Figures A and B). Considering the clinical history and MRI appearance, the diagnosis of submucosal hematoma secondary to barotrauma was made. Conservative treatment with oral decongestants and analgesics resulted in satisfactory symptom relief.

**Figure A:**
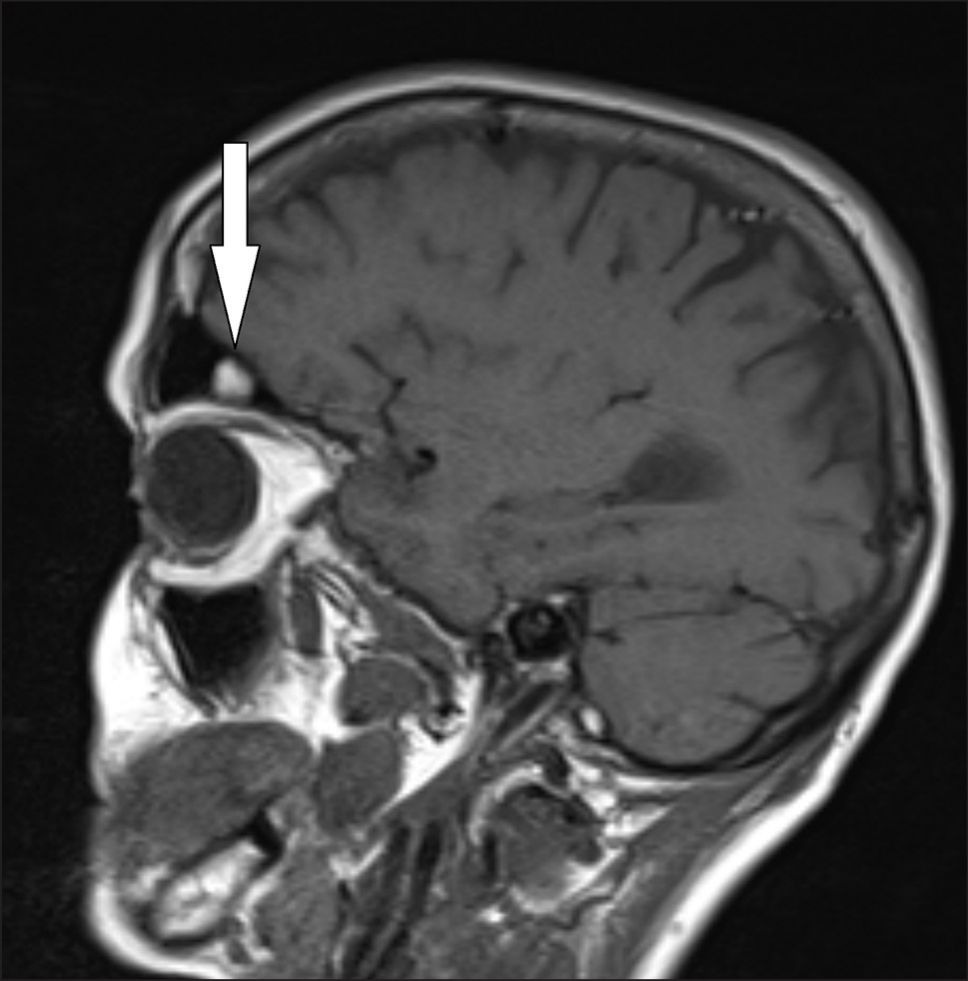


**Figure B:**
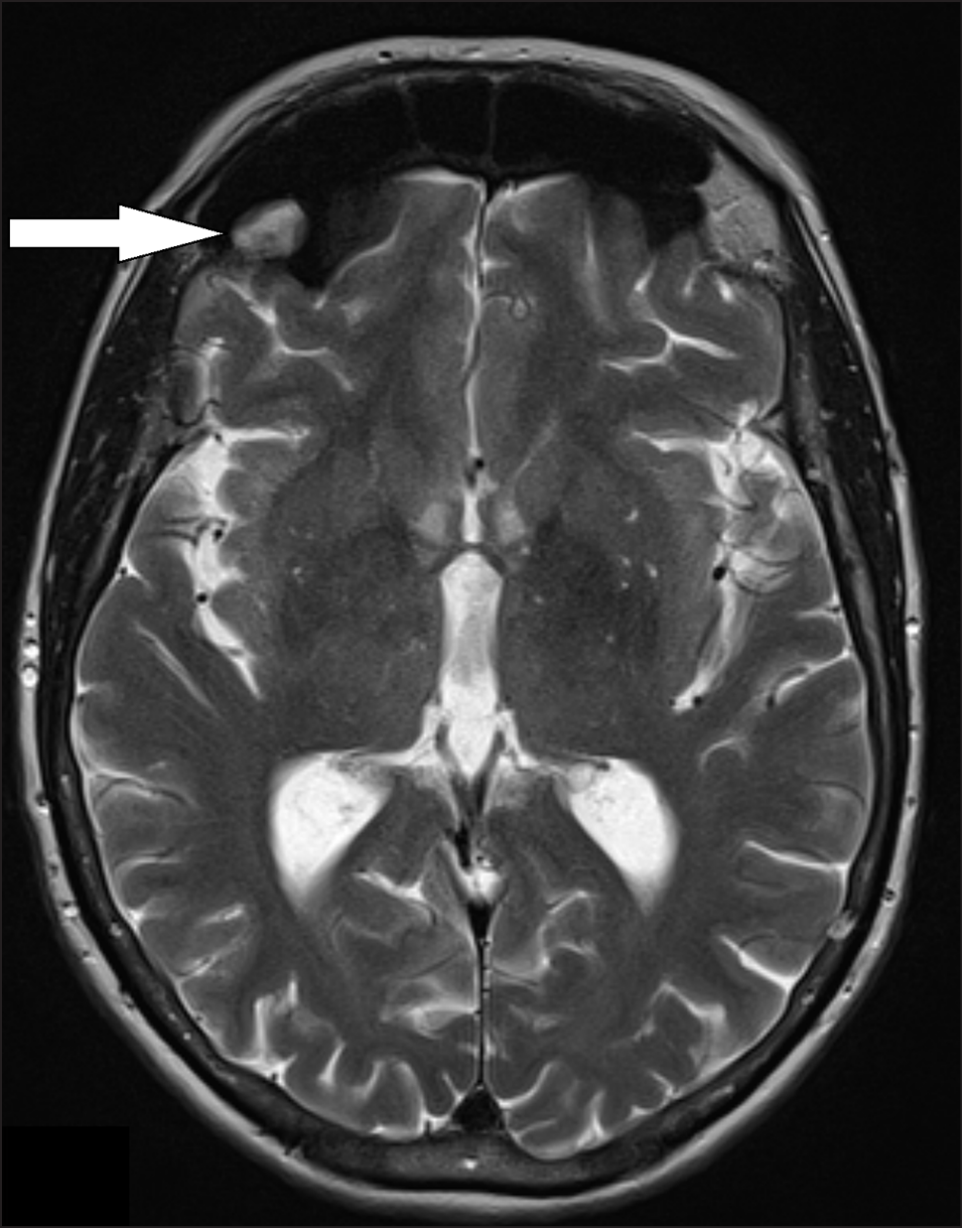


## Comment

Sinus barotrauma is a tissue injury caused by a rapid change in barometric pressure difference between the intrasinusal air and the surrounding atmosphere. This pathologic condition occurs most commonly in scuba divers and flight passengers subjected to abrupt pressure changes [1]. The pathophysiology of sinus barotraumas is related to Boyle-Mariotte’s law. It postulates the absolute pressure of an ideal gas being inversely proportional to the volume it occupies if the temperature remains unchanged within a closed system [2].

Normally, the ostium of each sinus opens into the nasal cavity, allowing equalization of air pressure. In our case, the tortuous and narrow right nasofrontal duct to the middle meatus in the nasal cavity was obstructed due to mucosal inflammatory changes illustrated on CT (Figure C). This inflammation was possibly initiated after an upper respiratory tract infection or allergic rhinitis. Other possible causes of obstruction include deviated nasal septum, nasal polyps, chronic sinusitis, neoplastic disease, or other anatomic deformities.

**Figure C:**
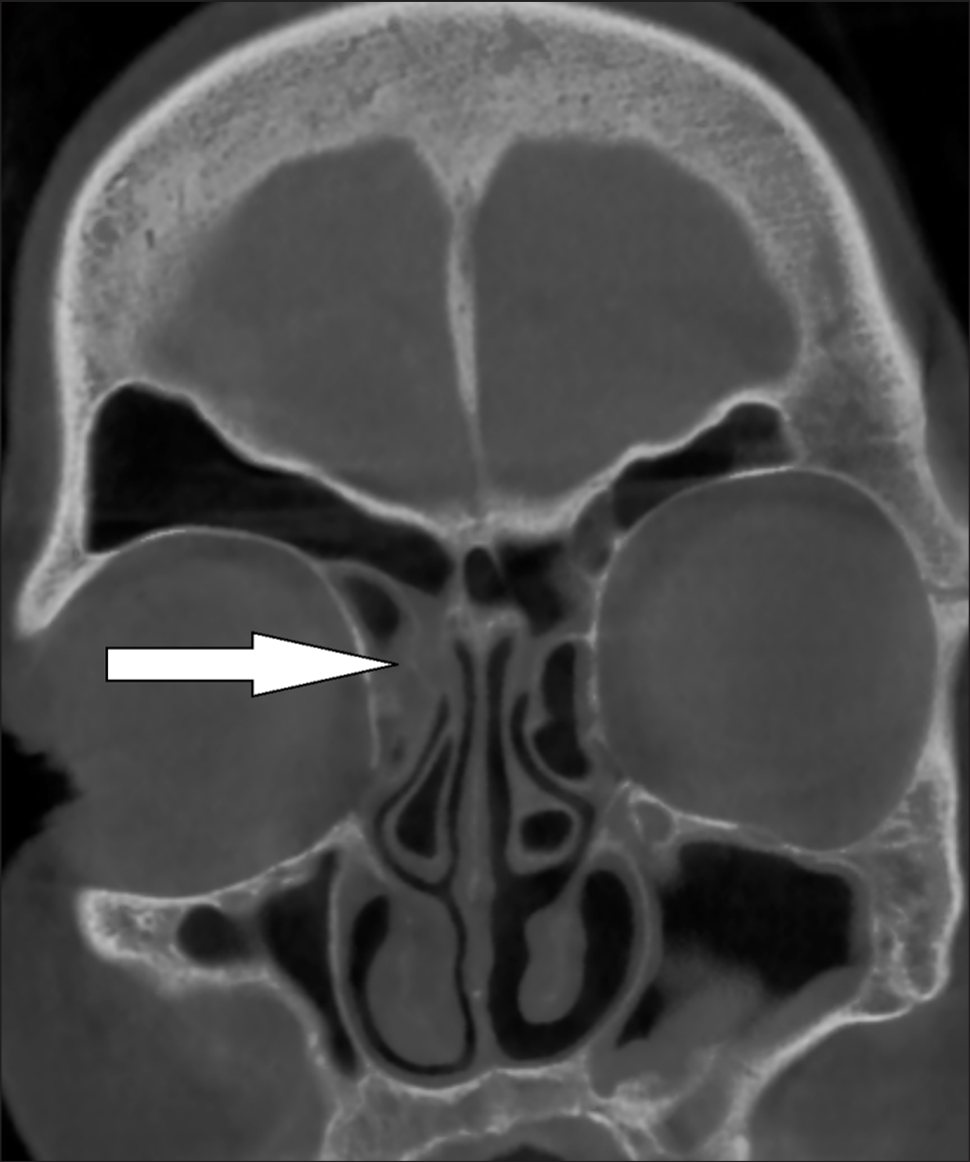


A blocked sinus ostium during a flight descent causes a relative negative intrasinusal pressure, resulting in mucosal edema, hemorrhage, or transudation of serosanguinous fluid to compensate for the decreased volume. Pain and epistaxis are the most common symptoms; rarely, neurological manifestations may occur due to irritation of the facial or infraorbital nerve. Differential diagnosis of a nonenhancing, T1 and T2 hyperintense polypoid sinusal mass include mucocele and exceptionally cholesterol granuloma. Absence of remodelling or erosion of the surrounding bone, clinical history, and spontaneous resolution of the mass favors submucosal hemorrhage secondary to barotrauma in our case.

Campbell and Weissman described three clinical grades of this entity. Grade I causes a mild transient sinus discomfort. Grade II is characterized by localized pain lasting up to 24 hours. Grade III, like in our case, exhibits severe pain lasting for more than one day. Treatment of barosinusitis is aimed at reestablishing sinus ventilation and decreasing mucosal inflammation with analgesics and nasal decongestants [1]. In recurrent moderate or severe cases with chronic discomfort, endoscopic sinus surgery may be required to enlarge the natural ostium to regain appropriate sinus ventilation. Frequent Valsalva maneuvers or prophylaxis with nonsteroidal anti-inflammatory drugs may aide in preventing sinus barotrauma in future flights.

## Competing Interests

The authors declare that they have no competing interests.
